# When communities are really in control: ethical issues surrounding community mobilisation for dengue prevention in Mexico and Nicaragua

**DOI:** 10.1186/s12889-017-4305-9

**Published:** 2017-05-30

**Authors:** Robert J. Ledogar, Carlos Hernández-Alvarez, Amy C. Morrison, Jorge Arosteguí, Arcadio Morales-Perez, Elizabeth Nava-Aguilera, José Legorreta-Soberanis, Dawn Caldwell, Josefina Coloma, Eva Harris, Neil Andersson

**Affiliations:** 1 CIET International, New York, NY USA; 2 CIET Nicaragua, Managua, Nicaragua; 3 Department of Entomology and Nematology, University of California, Davis, CA USA; 4 Centro de Investigación de Enfermedades Tropicales, Universidad Autónoma de Guerrero, Acapulco, Mexico; 5 CIET Canada, Ottawa, Canada; 6 Division of Infectious Diseases and Vaccinology, School of Public Health, University of California, Berkeley, CA USA; 7 Department of Family Medicine, McGill University, Montreal, Canada

**Keywords:** Camino verde, Socialisation of evidence, Community intervention research, Community mobilisation, Autonomy

## Abstract

We discuss two ethical issues raised by *Camino Verde,* a 2011–2012 cluster-randomised controlled trial in Mexico and Nicaragua, that reduced dengue risk though community mobilisation. The issues arise from the approach adopted by the intervention, one called Socialisation of Evidence for Participatory Action. Community volunteer teams informed householders of evidence about dengue, its costs and the life-cycle of *Aedes aegypti* mosquitoes, while showing them the mosquito larvae in their own water receptacles, without prescribing solutions. Each community responded in an informed manner but on its own terms. The approach involves partnerships with communities, presenting evidence in a way that brings conflicting views and interests to the surface and encourages communities themselves to deal with the resulting tensions.

One such tension is that between individual and community rights. This tension can be resolved creatively in concrete day-to-day circumstances provided those seeking to persuade their neighbours to join in efforts to benefit community health do so in an atmosphere of dialogue and with respect for personal autonomy.

A second tension arises between researchers’ responsibilities for ethical conduct of research and community autonomy in the conduct of an intervention. An ethic of respect for individual and community autonomy must infuse community intervention research from its inception, because as researchers succeed in fostering community self-determination their direct influence in ethical matters diminishes.

**Trial registration:** ISRCTN 27581154

## Background

In cluster-randomised controlled trials, especially when an intervention can impact all households in a cluster and the surrounding area, ethical issues arise concerning not only the rights of individual subjects but also the fact that these individuals live in the same neighbourhood and constitute a community.

Existing guidelines such as the Helsinki Declaration [1], the *Belmont Report* [2] and Canada’s *Tri-Council Policy Statement* [3] are mainly concerned with protection of individuals and their rights. Some guidelines speak of community participation in research. The Tri-council Statement has ethically nuanced special sections on research among indigenous communities. None of these manifests a consistent public health viewpoint.

Since the turn of the present century a growing number of voices have been arguing for adoption of a public health perspective in research ethics that takes into account not only risks and benefits to individual research participants but also those to the population as a whole [4–8] The United Kingdom’s Nuffield Council on Bioethics says that bioethical discussions should take ethical issues arising at the level of the *population* equally seriously and proposes a framework by which people can accept some personal restrictions in the interest of the wider population [9]. This framework is the “stewardship model”, meaning mostly government stewardship. In the United States, the American Public Health Association adopted a set of *Principles of the Ethical Practice of Public Health* that seek to achieve a balance between the traditional concerns of public health with respect for the rights of individuals with the common good [10], while proponents of community-based participatory research have expressed dissatisfaction with the narrow focus adopted by some ethical review boards and called for new guidelines that protect not only individual research participants but also communities and populations [11, 12].

## Purpose

Here we discuss two ethical issues arising within Camino Verde, a cluster-randomised controlled trial of evidence-based community mobilisation for dengue control and prevention in Nicaragua and Mexico. The intervention had a positive impact on serological evidence of dengue virus infection in children, reported illness at all ages, and all dengue vector control indices [13]. The two issues arise from a particular approach adopted in the intervention and they concern the tension between individual and community rights and the tension that arises between researchers’ responsibilities for ethical conduct of research and community autonomy in the conduct of an intervention.

In relation to the tension between individual and community rights, two ethical review boards for the Camino Verde trial questioned whether the intervention might lead to coercion and/or stigmatization of individuals.

The Oxford Dictionaries define coercion as the action or practice of persuading someone to do something by using force or threats [14]. Public health regulations such as quarantines, declaring certain locations to be smoke-free or requiring immunisations for entry into a country or a school, are coercive.

Erving Goffman famously called stigma “spoiled identity” [15]. Stigma links individuals to negative stereotypes, and stigmatisation can result in prejudice and discrimination [16].

Regarding the second tension, by community autonomy we mean that between researchers’ responsibilities for ethical conduct of research and community responsibilities such as those to recruit new volunteers and train them not only in the technical aspects of mosquito control but also in respectful treatment of residents and obtaining individual informed consent for household visits.

## The Feasibility Study

From 2004 to 2007, the Nicaraguan office of the CIET Group, an international non-governmental collection of researchers, conducted a feasibility study of community mobilisation for dengue prevention in the capital city of Managua [17]. This study developed four main strategic elements that would guide the *Camino Verde* intervention: the use of community volunteers, called *brigadistas*; house-to-house visits, called *visitas de acompañamiento*; visits to schools, churches, shops, clubs and other organizations; and a wide variety of collective events.

While training the brigadistas to educate their neighbours about the dengue virus and the behaviour of the *Aedes aegypti* mosquito, researchers were also learning about the ethical climate already prevailing in these communities where neighbours had learned over many years to live with one another and cooperate under conditions of high residential density with severe limitations on water supply and other public services. These communities had long experience finding their own collective solutions to day-to-day problems. From the collaboration of researchers and brigadistas conducting household visits together, there developed an ethic of respect that was to guide the conduct of the *Camino Verde* trial in which 19 brigadistas from the feasibility study became the facilitators who trained the brigades that would be the trial’s main driving force.

## The Trial

The *Camino Verde* trial was a collaborative effort between researchers at the University of California, Berkeley, two member organizations of the CIET Group - the *Centro de Investigación de Enfermedades Tropicales* (CIET) at the University of Guerrero in Acapulco, Mexico and CIET in Nicaragua - together with 150 neighbourhoods: 60 of them in the Nicaraguan Capital, Managua, and 90 in three coastal regions of Guerrero state in southwest Mexico [13]. Data collection was limited to clusters of some 140 households in each neighbourhood but the intervention activities often extended beyond the cluster boundaries.

The baseline (August 2010–January 2011) and follow-up impact (August 2012–January 2013) surveys each included an entomological survey, collection of paired saliva samples before and after the dengue season to detect recent dengue infection, and questionnaires related to dengue, social capital and costs.

### Approach

The mobilisation approach is called Socialisation of Evidence for Participatory Action (SEPA) [18].

As long ago as 1951, Lewin argued that the process of ‘unfreezing’ existing behaviour patterns needs to take place in a group environment and to involve open and supportive communication among those involved in negotiating the change [19]. Parsons maintains that if a system is to make a significant change from its status quo, the changes are likely to come from creative self-organizing rather than from planned change [20]. Hawe, Trickett and others propose thinking of interventions as events in systems that either leave a lasting footprint or fade away depending on how well the dynamic properties of the system are harnessed [21, 22]. The SEPA approach does not seek individual behavioural change in and of itself, *but participatory action leading to change* at household, community, municipal and national levels. Risk communication is often used for sharing evidence, but not to prescribe specific courses of action. CIET “socialises” evidence for community members to respond to it in light of their own reality, in an informed manner but on their own terms, which often implies working out conflicting views and interests in any given society. As the autonomous community action process gains strength, the research team reduces its facilitator role, aiming to promote sustainable self-management beyond the intervention period.

### Actors

In *Camino Verde*, key roles in implementing the SEPA approach were played by:

The *brigadistas*. These mobilisers and educators constituted the backbone of the effort. All were residents of the communities where they conducted SEPA activities and all had to be acceptable to other community members. Facilitators (see below) trained them in the life-cycle and habits of the *Aedes aegypti* mosquito and the dengue virus transmission cycle and assured that brigadistas communicated respectfully with householders and other community members. Brigadistas learned their roles by accompanying facilitators in making initial contact with households. Volunteers who joined brigades after the initial contacts were usually trained by other brigadistas.

The *facilitators*. The facilitators’ role was to (1) make initial contact with the community and facilitate a brigadista recruitment process, (2) present evidence, (3) provide training, (4) support the community in its assuming of responsibility for the intervention. In Nicaragua, the facilitators were former brigadistas active in the 2004–2008 feasibility study on the same subject. Facilitators in Mexico, mostly recent graduates from the University of Guerrero where CIET is located, received more formal ethical training in which a Mexican communications expert and a member of the Nicaraguan field team participated. In both countries, facilitators sought to move as quickly as possible from leadership roles to supporting ones.

The *households*. Environmental control of the dengue mosquito at household level was indispensable to the entire effort. All consenting households in the research clusters participated in the intervention and all members of each household were invited to join in the effort. While measurement was limited to the approximately 140 households in the cluster, the intervention often reached households in the surrounding neighbourhood as well.

*Community leaders*. The Nicaraguan trial was entirely concentrated in the capital city, Managua, where neighbourhoods typically have recognised, active leadership closely allied with the Sandinista government. The SEPA strategy there was to work with these leaders and deliberately avoid creating parallel structures, while striving to maintain the brigade’s autonomy and political neutrality. Several brigadistas were also community leaders. The Mexican trial covered the entire coastal area of the State of Guerrero. In Guerrero’s rural areas, the strategy was similar to that in Nicaragua, especially where the communities are primarily indigenous and more organized. In urban areas, mainly in the city of Acapulco, identifiable community leadership tended to be less unified and less effective for our purposes. The organization of many urban communities has been disrupted by violence and the *Camino Verde* brigades in some cases helped to restore community structure.

*Other organizations*. Numerous national and regional organisations in both countries, while not rooted in any individual community, are active at the grassroots level. These organisations had diverse main agendas but the threat of dengue and the need for mosquito control was a common concern. The SEPA programme partnered with as many of these as possible in its mobilisation activities.

In both countries government health authorities approved of the trial and were kept informed about its progress.

### *Key activities*

These included:

*Household visits* (called *visitas de acompañamiento*). During these visits, brigadistas explained the development-cycle of the dengue virus-transmitting mosquito and enlisted householders in the control effort. The *Aedes aegypti* mosquito develops in clean water found in barrels, jugs, washtubs, flowerpots, planters and other household containers. Once pointed out, the mosquito larvae and pupae are recognisable. In Managua and the coastal regions of Mexico’s Guerrero State, many households must store water because they have no steady piped-water supply.

*Discussion groups* to elicit informed community consent. In Nicaragua, the intervention was launched by a series of discussions with community leaders about the costs of dengue and its control [23]. In Mexico, discussions with selected residents centred around ways of organising the community and involving all the relevant actors.

*Community-wide publicity and mobilisation tools*. These included songs, games, sports, murals, graffiti, t-shirts, bracelets, street theatre and clean-up campaigns to collect and dispose of empty containers, used tires and other repositories of water where mosquitoes may breed. In Nicaragua a blogging site was available where community members shared experiences.

*Community-specific strategies*. In many cases, communities invented or identified their own strategies based on local circumstances. For example, in some rural Mexican communities, local fish species were known to control mosquitoes so the community developed a distribution scheme for these fish [24]. Dealing with egg-laying sites in common spaces required solutions tailored to the nature of the sites (cemeteries, plazas, playgrounds and playing fields, waste disposal sites, bus stations, businesses, etc.) and usually required liaison of communities with various public services.

*Neighbourhood peer monitoring*. Aside from the work in their own neighbourhoods, brigades also engaged in formal peer monitoring and evaluation, with a brigade from one neighbourhood monitoring the work of its counterpart in another neighbourhood. At the end of each neighbourhood peer visit, the visiting team processed the results on site and shared the evidence with the host brigade and community leaders. Among other effects, this helped to assure a certain consistency in the way brigades approached their tasks, both technically and ethically.

*Meetings with and among brigades*. Over the course of the intervention there were periodic meetings among brigades from different communities in which researchers also participated. This often took the form of activities in one neighbourhood to which brigades from other neighbourhoods were invited. It was easier to arrange such activities in the urban areas of Managua and Acapulco but next to impossible in the more widely dispersed communities of the Costa Grande and Costa Chica of Guerrero, Mexico.

### Ethical Review

Although the Mexican and Nicaraguan interventions were two arms of the same trial, the Nicaraguan arm was funded through the University of California at Berkeley (UCB) via New York-based CIETinternational, whereas the Mexican arm received its support through CIETcanada. Thus, five separate ethical review processes were required: for Nicaragua, the Institutional Review Boards (IRBs) of UCB (approval 22 July 2010 with annual reviews) and CIETinternational (approval 1 August 2010 with annual reviews) plus the Nicaraguan Ministry of Health (approval 25 August 2010 with annual reviews); for Mexico the Ethics Committee of the Tropical Disease Research Institute at the *Universidad Autónoma de Guerrero* (approval 27 November 2009) and the Research Ethics Board (REB) of CIETcanada (16 November 2009 with annual reviews).

The issue of possible coercion was raised by CIETinternational’s IRB in the Nicaraguan case and by CIETcanada’s REB with reference to Mexico. Both boards asked how the researchers intended to deal with the potential problem of stigmatisation of households that do not wish to participate in the mosquito control activities or have difficulty participating (e.g., as a result of poverty). Investigators responded as follows:

- Dengue is a public health problem involving both individual rights and those of the wider community. Households and small businesses that refuse to do anything to control mosquito breeding sources on their property may be endangering not only themselves but surrounding properties as well.
- Camino Verde is no more coercive than current government chemical control efforts, involving treatment of household water containers with a chemical larvicide (temephos) and space spraying to control adult mosquitoes.
- In the *Camino Verde* approach, residents *are* free not to participate, but the team hoped that those who control mosquito development on their own premises and know that they could still be infected by mosquitoes from nearby premises would put some kind of pressure on any non-participant neighbours.

- The SEPA brigades do make use of social pressure but combine it with neighbourly cooperation. Examples from the Managua pilot study offered to the IRB included the following:
  a) Families that live by occupations requiring long absences from home and irregular hours: SEPA brigadistas discussed controlling household water receptacles with these families to identify whether one family member could devote some time regularly to this task or different ones could take turns. Neighbours, or even other brigadistas were sometimes recruited to assist.
  b) Households composed of single working women with small children and households with only elderly occupants: in these cases, stronger emphasis was placed on obtaining cooperation/assistance of neighbours and motivating the latter by way of their self-interest in eliminating a neighbouring source of contamination.
  c) Households inhabited by persons capable of performing regular maintenance activities but refusing to do so: in these cases, brigadistas attempted to bring these people together with their neighbours to discuss the issue.
  d) Small businesses like scrap dealers and auto repair shops whose owners refuse to take any measures to control breeding sites on their properties: when efforts to persuade business owners to be more cooperative failed, such businesses were reported to health authorities as a menace to the rest of the community.

The two review boards accepted these responses and responses to other queries and gave clearance for the interventions to proceed.

### Developments over time

At the start, the Mexican facilitators were the de facto managers of the intervention in each neighbourhood. The brigadistas gradually assumed more prominent roles, but the facilitators remained in frequent contact with the communities until the trial’s end.

While in Mexico, brigadistas were paid a small “incentive”, in Nicaragua the community leadership issued a call for volunteers. A number of the most active brigadistas also held positions of responsibility within larger community organizations. In late 2011, a system was put in place whereby funds provided by CIET for training, transport, refreshments at special events, etc., instead of being managed by the facilitator, went directly to the coordinator of the neighbourhood brigade who accounted for them each month both to the community leadership and to CIET. At the beginning of 2012 the facilitators began to withdraw from day-to-day involvement in their respective communities. Community “ownership” of the intervention was thus more advanced at the trial’s end in Nicaragua.

As community autonomy in the conduct of the intervention increased, researchers had less leverage to exert in the ethical sphere. Brigadistas, all of them volunteers and many of them quite young, came and went. New brigadistas were trained by other brigadistas instead of by facilitators. A smaller team of former facilitators still visited the communities regularly and discussed issues of importance with brigades and the leadership; one of their tasks was to make sure that values of respect for individual autonomy were maintained and reinforced. Community peer monitoring, as mentioned above, also helped maintain some consistency in the way that brigades approached communities. Nevertheless, the research team’s overall influence over the ethical conduct of the intervention became less and less direct.

## Philosophical issues

Among numerous philosophical positions one might adopt in this case, three are most commonly contrasted: utilitarian, liberal (Kantian) or communitarian [6, 25]. The responses to the ethics panels reported above would appear to have most in common with a communitarian perspective. And indeed one might see in the Nicaraguan experience a reflection of Charles Taylor’s thesis that the essential condition of a non-despotic regime is that citizens have a patriotic identification with the political regime as a common enterprise in search of the common good [26]. But adopting a communitarian perspective does not free researchers and community leaders from the obligation to respect individual autonomy and seek informed consent.

David Buchanan cites Jurgen Habermas in arguing that people have a right not to be bound to norms other than those to which they give un-coerced rational consent, and that it is only by engaging in dialogue with others that one can become convinced of the validity of any proposed norm. Justice consists in permitting all persons to participate freely and equally in conversations aimed at reaching consensus on norms regulating conduct [27].

The SEPA field team in Nicaragua discussed the interaction between brigadistas and other residents in terms of just such conversations:

Every SEPA dialogue takes place around certain concrete bits of evidence that lead to very different interpretations. Actions are reviewed, new knowledge is produced and alternatives are sought as to what should be done and how -- seeking all the while to achieve consensus on the basis of free and informed decisions as to what can be done with the resources at hand. Through daily actions like these, learning occurs and an ethic of respect for others and their self-determination is built. --*From an internal blog maintained by Nicaraguan investigators, facilitators and brigadistas* [28].

Nevertheless, there are situations where community organisations are unable to engage in such a dialogue. This occurs especially with local businesses (stores, repair shops, scrap dealers, small factories, etc.) whose owners are inaccessible or uncommunicative. When all efforts at dialogue are exhausted, community leaders can and do call upon authorities to oblige the owners to control receptacles on their properties that are sources of contamination.

We believe that community mobilisation interventions such as the SEPA approach described here could become excessively coercive if they are not conducted with sensitivity and respect for individuals. On the other hand, individuals have a duty to avoid putting their neighbours at risk by failing to take the simple measures necessary to prevent mosquitoes from breeding on their premises. In the APHA *Principles of the Ethical Practice of Public Health* there is a comment on the second principle about the common need in public health to weigh the concerns of both the individual and the community. It states that there is no ethical principle that can provide a solution to this perennial tension in public health [10].

If handled properly, such tension between individual and community rights over a fairly straightforward issue such as dengue control and prevention could become creative in setting a pattern for resolving other issues in the same communities and thereby contribute to building positive social capital among residents. If not handled properly, this tension could indeed lead to less positive conditions.

We cannot be certain that the structures we have supported or helped to create will always and only be beneficial to all. We wanted neighbourhoods to take on greater responsibility for dengue control and we hoped that the collective effort required might serve as a catalyst for their working together to solve other problems as well. But the more success we had in encouraging neighbourhoods to pursue their own green way to dengue control the less influence we, as researchers, had over the ethics of the outcome.

That is why the initial formation of facilitators, and theirs of brigadistas, was crucial for setting the ethical standard for the entire trial and beyond.

## Conclusion

The SEPA approach involves partnerships with communities, presenting evidence in a way that brings conflicting views and interests to the surface and encourages communities themselves to deal with the resulting tensions. One such tension is that between individual and community rights. This tension can be resolved creatively in concrete day-to-day circumstances provided those seeking to persuade their neighbours to join in efforts to benefit community health do so in an atmosphere of dialogue and with respect for personal autonomy.

An ethic of respect for individual and community autonomy must infuse community intervention research from its inception, because as researchers succeed in fostering community self-determination their direct influence in ethical matters diminishes.

## Abbreviations

APHA: American public health association
IRB: Institutional review board
REB: Research ethics board
SEPA: Socialisation of evidence for participatory action
UCB: University of California, Berkeley
